# Considerations for Quality Control Monitoring of Machine Learning Models in Clinical Practice

**DOI:** 10.2196/50437

**Published:** 2024-06-28

**Authors:** Louis Faust, Patrick Wilson, Shusaku Asai, Sunyang Fu, Hongfang Liu, Xiaoyang Ruan, Curt Storlie

**Affiliations:** 1 Robert D and Patricia E Kern Center for the Science of Health Care Delivery Mayo Clinic Rochester, MN United States; 2 Department of Artificial Intelligence and Informatics Mayo Clinic Rochester, MN United States

**Keywords:** artificial intelligence, machine learning, implementation science, quality control, monitoring, patient safety

## Abstract

Integrating machine learning (ML) models into clinical practice presents a challenge of maintaining their efficacy over time. While existing literature offers valuable strategies for detecting declining model performance, there is a need to document the broader challenges and solutions associated with the real-world development and integration of model monitoring solutions. This work details the development and use of a platform for monitoring the performance of a production-level ML model operating in Mayo Clinic. In this paper, we aimed to provide a series of considerations and guidelines necessary for integrating such a platform into a team’s technical infrastructure and workflow. We have documented our experiences with this integration process, discussed the broader challenges encountered with real-world implementation and maintenance, and included the source code for the platform. Our monitoring platform was built as an R shiny application, developed and implemented over the course of 6 months. The platform has been used and maintained for 2 years and is still in use as of July 2023. The considerations necessary for the implementation of the monitoring platform center around 4 pillars: feasibility (what resources can be used for platform development?); design (through what statistics or models will the model be monitored, and how will these results be efficiently displayed to the end user?); implementation (how will this platform be built, and where will it exist within the IT ecosystem?); and policy (based on monitoring feedback, when and what actions will be taken to fix problems, and how will these problems be translated to clinical staff?). While much of the literature surrounding ML performance monitoring emphasizes methodological approaches for capturing changes in performance, there remains a battery of other challenges and considerations that must be addressed for successful real-world implementation.

## Introduction

As machine learning (ML) models integrate into clinical practice, ensuring their continued efficacy becomes a critical task. A pervasive limitation in ML is the inability of most models to adapt to changes in their environment over time. As a result, a model that may have performed exceptionally in its development environment can become gradually or immediately less accurate while in production [1,2]. This problem has been well studied by the ML community, with current literature offering invaluable methodological strategies for the detection of declining model performance and the ethical implications of such declines [3-7]. However, the proper choice of monitoring algorithm is only one step in the larger series of problems and considerations surrounding the sustained maintenance of these models in a real-world scenario. While some authors address the wider set of problems encountered in the long-term maintenance strategy of a deployed model, it is typically only an acknowledgment of these problems, rather than the personal experiences and solutions developed to solve them [8,9]. As such, we aimed to supplement current literature with an alternative approach in which we provided an in-depth review of the experiences and challenges encountered when integrating our ML monitoring solution into clinical practice.

This paper focuses on an ML model implemented into Mayo Clinic’s practice in 2018. The model, known as “Control Tower,” is a fully integrated health care delivery model that predicts the need for inpatient palliative care through modeling palliative care consultation. The model runs automatically on all inpatients at Mayo Clinic’s St Marys and Methodist Hospitals in Rochester, Minnesota, with patient scores monitored by the palliative care practice [10]. The approach was to treat the palliative care consult as a time-to-event outcome. Some of the features used are static (patient demographics and prior history), while others are time varying or dynamic (such as laboratory values, vitals, and in-hospital events). To capture the time-varying nature of these covariates, we used a heterogeneous Poisson process. Furthermore, it was crucial to account for nonlinearity and interactions; as a result, we used a gradient boosting machine. The model was validated through a clinical trial conducted from 2019 to 2022 to assess real-world effectiveness and is still in use by the palliative care practice as of July 2023 [11,12]. The study by Murphree et al [10] provides a complete methodological overview of the ML model and validation procedure. The Control Tower monitoring platform was developed and implemented over the course of 6 months. The platform has been used and maintained for 2 years and is still in use as of July 2023.

This paper provides a series of guidelines for developing and integrating ML performance monitoring into a team’s workflow. Guidelines were developed from real-world experiences and challenges encountered throughout this process by a data science team at Mayo Clinic. In addition, a comprehensive overview of the developed monitoring platform is provided, as well as the accompanying source code for demonstration purposes (Multimedia Appendix 1). Overall, this paper serves as a primer for considerations that must be made when implementing and maintaining a model-monitoring system in a clinical setting, coupled with the corresponding solutions that our team had used.

## Development of the Model Monitoring Platform

### Overview

Traditionally, guidelines are developed through expert-driven processes, such as the Delphi method that seeks to provide standards through initial conceptions followed by several rounds of revisions until ultimately converging to an agreed-upon set [13]. However, in emerging areas where expertise is sparse, expert-driven approaches are often costly when seeking consensus of multiple experts through multiple rounds of responses [14]. An alternative to the expert-driven approach is experience-driven methodologies, which emphasize the personal experiences and observations of individuals who have directly encountered the phenomena. Normally these methodologies focus on practical knowledge through the explication of the “real world.” Our team opted to derive a set of guidelines based on our specific real-world experiences and the challenges faced when designing, implementing, and integrating the Control Tower monitoring platform. Our specific methodologies used throughout this process are documented here and later generalized into a series of guidelines in the *Design Considerations* section.

### Establishing the Team and Responsibilities

When planning the phases of Control Tower, it was decided that the role of monitoring the model would remain with the model development team. The task of monitoring was divided among 4 team members, rotating the responsibility of monitoring, monthly. This approach ensured monitoring would not significantly inhibit the bandwidth of any 1 team member. Monitoring responsibilities did not fall to the team member who developed the model, as their primary task in monitoring would be to retrain the model when necessary. The monitoring platform was checked biweekly, Mondays and Thursdays, to balance coverage and analyst time. The Monday check ensured immediate response to any issues that may have occurred during the previous weekend, and the Thursday check provided enough time before the upcoming weekend to identify and resolve any errors that may have occurred during the week. Typically, a single-model monitoring session would take approximately 5 to 10 minutes, assuming no problems were encountered.

### Platform Development

#### Overview

Performance monitoring of Control Tower was accomplished through the development of an R Shiny web application that comprised data visualizations and interactive tables. The goal was to create a centralized, user-friendly platform for all team members to check model performance. The platform consisted of 5 different tabs addressing different types of data shift, providing multiple degrees of granularity depending on the depth of investigation required. The model used a set of 126 features, measured daily, and was called an average of 80,000 times per day. Daily metrics collected for performance monitoring included mean and scale covariate shifts per feature, predicted probabilities, and the number of daily predictions made by the model. The resulting data size of these collected performance monitoring metrics was trivial; however, capturing patient-generated data resulted in data creation on the order of GB per day, requiring a dedicated storage space.

**Figure 1:**
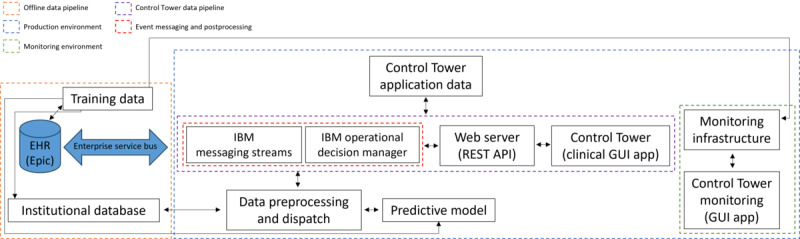
System architecture for Control Tower. For the Control Tower monitoring platform, we have 3 parent processes (training, production, and monitoring) that constitute our deployment. Child processes include the orchestration of the streams, events, and the prediction pipeline, which sends scores to the graphical user interface (GUI). EHR: electronic health record; REST API: Representational State Transfer application programming interface.

Figure 1 provides an overview of the system architecture for the Control Tower model and monitoring platform. The figure details the offline data pipeline used for the initial training of the Control Tower model; the components of the broader production environment and pipelines necessary for the predictive model and clinical graphical user interface (GUI) app; and finally, the components necessary for monitoring the performance of the Control Tower model. A more detailed visualization and comprehensive description of the system architecture is provided by Murphree et al [10]. Briefly, they outlined our deployment strategy which integrates a Representational State Transfer application programming interface within a Docker container, enabling the integration of predictive models into the Control Tower GUI. The data ingestion and preprocessing pipeline, integrated with IBM Streams and Operational Decision Manager, facilitates real-time prediction processing triggered by updates to institutional health records (Health Level Seven messages by our electronic health record). The Control Tower GUI application is built with Angular (Google LLC).

#### Monitoring Model Probabilities

**Figure 2:**
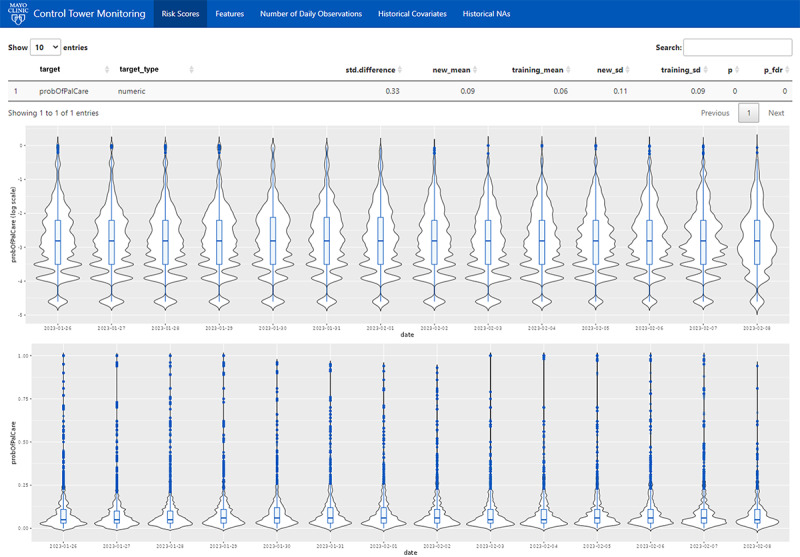
The startup screen of the Control Tower performance monitoring platform. This screen provides the user a quick overview of the model’s predicted probabilities over the past 2 weeks. The table near the top provides several statistics comparing the distribution of predicted probabilities over the last 2 weeks with the predicted probabilities on the training data. The 2 graphs contain a series of violin plots featuring the daily distribution of predicted probabilities. Given that the predicted probabilities cluster near 0, the distributions are also displayed on the log scale for easier visual inspection.

In the absence of ground truth labels, predicted probabilities from the model were monitored as an alternative to evaluating model performance. The platform visualizes these predicted probabilities as distributions of daily risk scores (Figure 2). Distributions are plotted on probability and logarithmic scales, allowing for easier detection of shifts when most predicted probabilities are low, considering that most patients will not be “high risk.” Historical daily distributions extend back 2 weeks, which is considered an optimal amount of time to notice shifts without overwhelming the user with data. Alongside these visualizations, several statistics are presented for comparing the prior 2 weeks data against the original training data. Means and SDs for the incoming and training distributions, the standard difference, and a *P* value for the Kolmogorov-Smirnov test of differences between the 2 distributions are provided. These statistics allow the user to detect gradual, more long-term changes that may go unnoticed when surveying 2 weeks of historic data. Overall, the tab shown in Figure 2 provides an overview of model predictions, allowing the user to quickly gauge whether a sudden or gradual probability shift has occurred.

#### Monitoring Covariate Shift

**Figure 3:**
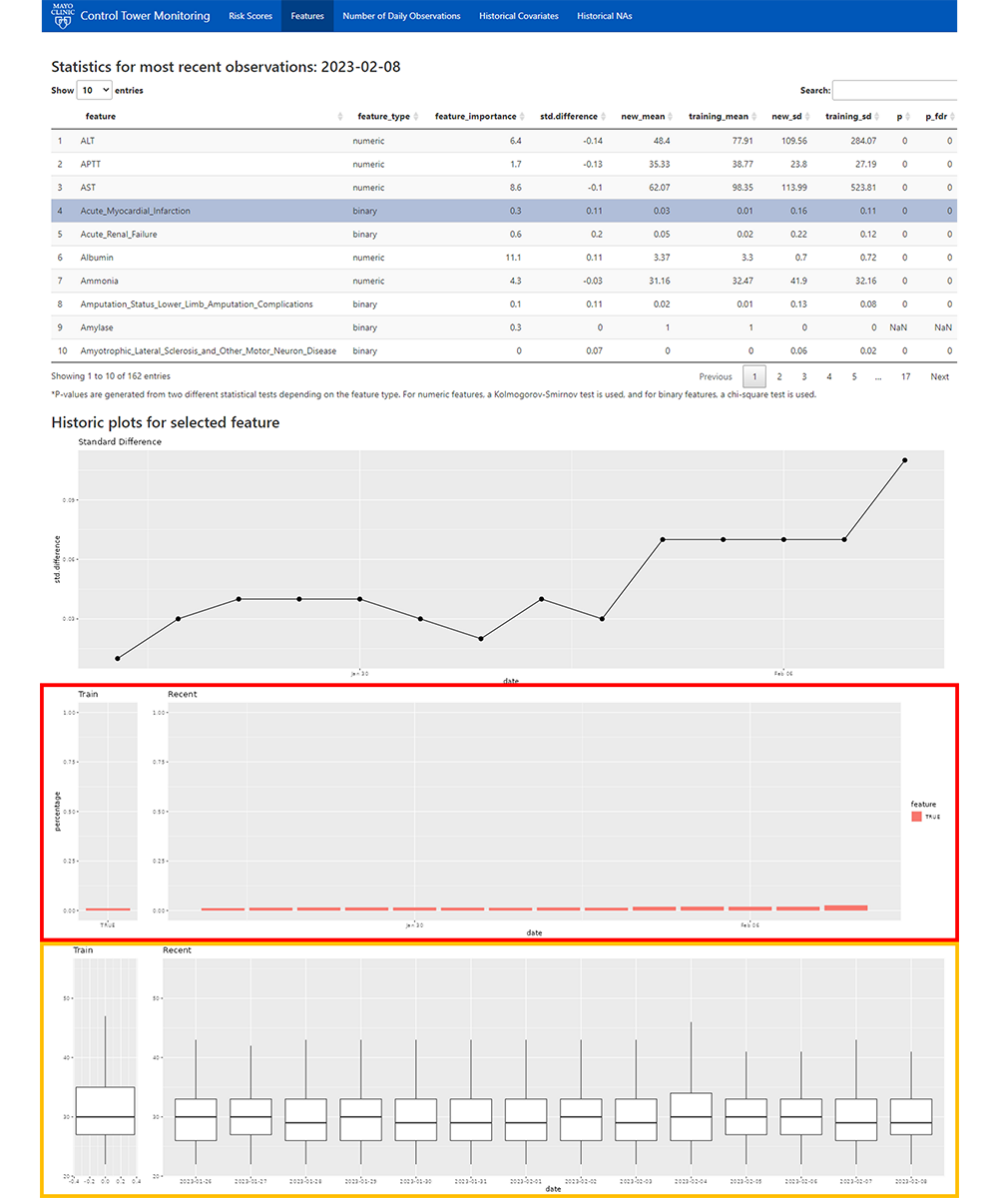
The “Features” screen of the platform details the distributions of all features used by the model. The distribution of each feature based on the last 2 weeks of data is compared with the feature’s distribution from the training data. These comparisons are provided via statistics in the table near the top, which can be sorted by each statistic to quickly find features with potential drift. Clicking on a feature populates 2 graphs, which are displayed below the table. The first graph displays the standardized difference between the feature’s distribution for that day against the distribution from the training data. Below this graph, one of the 2 graphs will be displayed depending on whether the selected feature was binary or continuous. These graphs display the daily distributions of the feature, using bar graphs for binary features (red outline) or box plots for continuous features (yellow outline).

Covariate shift was addressed in Control Tower by creating an interactive table containing all features included in the model (Figure 3) [4]. The table lists feature names and type, that is, continuous or discrete, and displays different statistical tests and plots, dependent on the feature type. To assess the impact of a feature with drift, the team included global feature importance scores from the originally trained model, in this case, the gradient boosting machine’s relative influence rank statistic. Providing a ranking of features based on the extent of error reduction in the model enables the user to triage different drifts. All other things being equal, a drifting feature with higher importance to the model than another feature would indicate a higher priority need of a fix. Similar to the predicted probability tab, the previous 2 weeks of incoming data are compared with the training data, with standard differences, means, and SDs provided. To accommodate for the discrete variables present, the distributional Kolmogorov-Smirnov test is changed to the chi-square test. The user can sort the table by column, allowing them to quickly pinpoint features, for example, with high standard difference. Clicking on a feature’s row in the table generates 2 plots underneath the table: the first is a line graph visualizing the daily standard differences, spanning back 2 weeks, and the second plot is dependent on the feature type. For continuous variables, the plot compares the feature’s daily distributions over the past 2 weeks with the distribution of the training data, using box plots. For discrete variables, bar plots are displayed in a similar fashion indicating the percentage of patients where the discrete feature was present or “True.” In tandem with the interactive table, these plots provide an efficient means of investigating a feature’s historic values at a glance.

**Figure 4:**
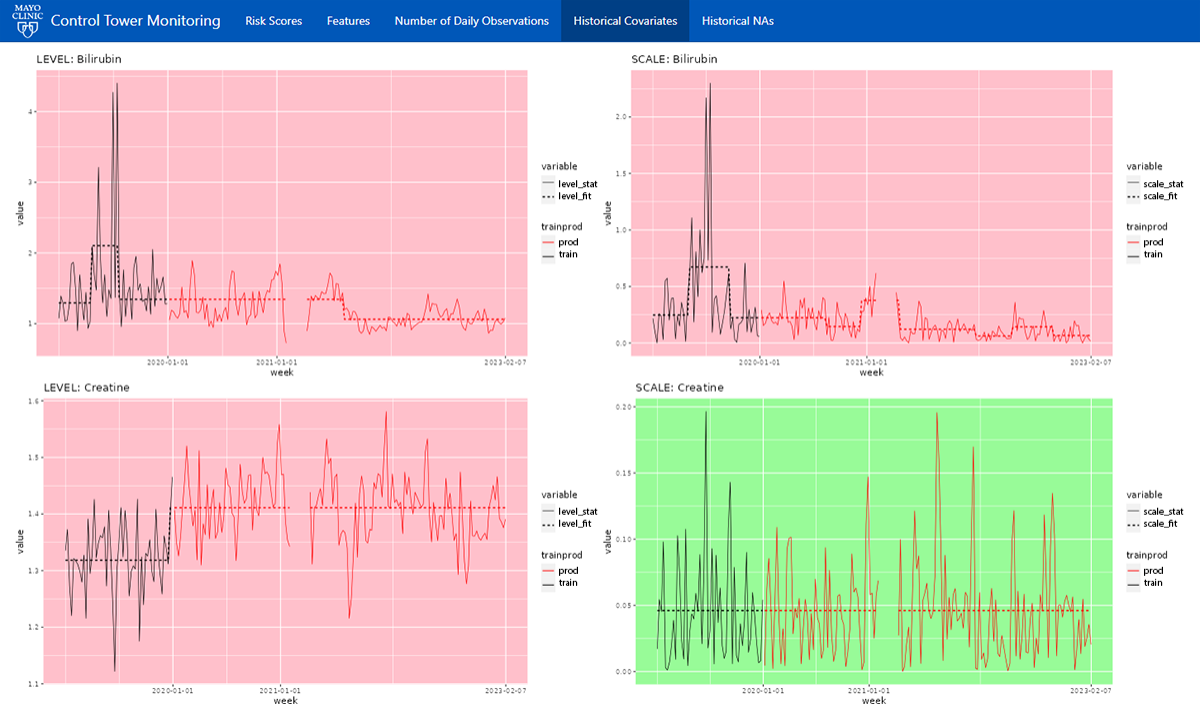
The “Historical Covariates” screen of the platform visualizes each feature’s daily distribution, beginning with the training data and then spanning from the day the model was deployed and onward. Each feature contains plots for the location (level) and dispersion (scale) of the nonparametric distribution. Each feature’s graph is color-coded “green” or “red” to indicate whether the feature’s distribution has significantly drifted from the initial training distribution, with red indicating significant drift.

**Figure 5:**
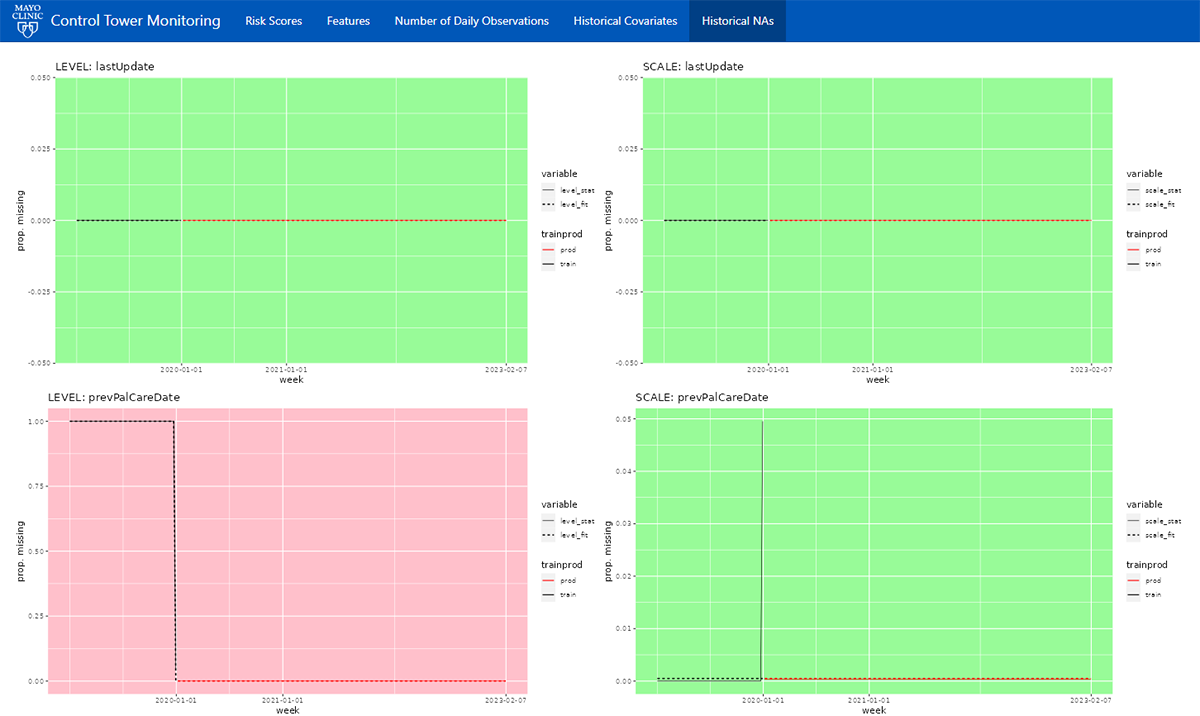
The “Historical NA’s” screen of the platform visualizes each feature’s historical missingness, beginning with the training data and then spanning from the day the model was deployed and onward. Each feature contains plots for the location (level) and dispersion (scale) of the nonparametric distribution. Each feature’s graph is color-coded “green” or “red” to indicate whether the feature’s missingness has significantly drifted from the initial training distribution, with red indicating significant drift. NA: not available.

When a deeper investigation into a feature is necessary, the 2-week “look-back” may be insufficient. Therefore, the platform also keeps a log of the full historic feature trends, spanning back to when the model was deployed (Figure 4). Feature plots are sorted by the model’s global feature importance and color-coded “green” or “red” to indicate whether the feature significantly drifted from the initial training distribution. Significance was determined via a nonparametric test developed by Capizzi and Masarotto [15], using a *P* value of .05. A nonparametric model was used because a moderate number of features were highly skewed, making traditional methods that assume normal distributions unworkable. Each feature contains plots for the location (level) and dispersion (scale) of the distribution. Overall, this tab, in addition to serving as a historical reference, provides a simple way to spot check for gradual shifts. Finally, an additional tab (Figure 5) is provided to assess the proportion of missing values over time, using the same visualizations and tests.

**Figure 6:**
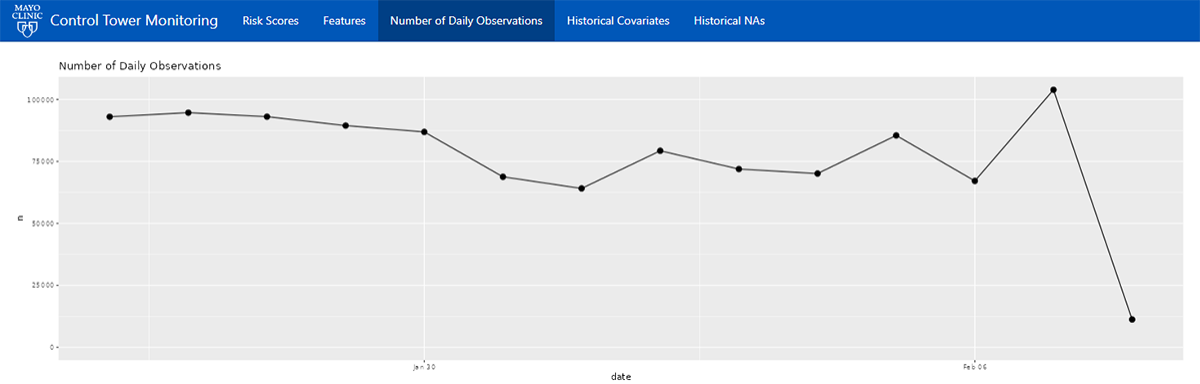
The “Number of Daily Observations” screen visualizes the number of model calls or predictions made each day over the past 2 weeks.

The final tab of the platform provides a simple line graph displaying the number of daily calls made to the model within the previous 2 weeks (Figure 6). Monitoring the number of daily calls can provide quick insight into whether the model is performing appropriately. For example, an abnormal number of model calls in a day, such as 0, may indicate an error in the data pipeline or model environment.

### Error Classification

Any production-level model is susceptible to various errors, and Control Tower was no exception. Most errors primarily revolved around technical infrastructure, particularly issues with databases being inaccessible due to nightly processing or a high influx of requests. In Textbox 1, a sample of encountered errors while monitoring Control Tower is presented. Although some errors were seemingly random occurrences, such as server reboots or expired certificates, others were more frequent and persistent. For instance, every night at specific hours, the database that supplies data to Control Tower, called Clarity, became unresponsive due to data updates. On January 5, 2022, this process was delayed and caused errors in the morning scores. In addition, updates to our electronic health record (Epic, Epic Systems Corporation), often resulted in Clarity being temporarily unavailable. In such cases, most issues were resolved on the same day, requiring no further action besides acknowledging the possibility of outdated or missing scores. However, a few errors necessitated intervention. On November 7, 2022, a data mart containing diagnosis codes underwent structural changes, breaking a Control Tower query. Furthermore, the team identified a covariate shift where they observed a gradual decrease in troponin blood tests. This error was traced back to a change in laboratory codes used for troponin; the clinical practice had adopted a new laboratory code that was not present in the training data. To address this, the error was rectified by associating the new codes with the “Troponin” feature on the platform’s back end.

**Error logging: A convenience sample of encountered errors while monitoring Control Tower is presented. This was constructed through email chains of discussions between IT personnel who oversaw the Control Tower system and the data scientists who oversaw model delivery.**

**Date and error**

- August 26, 2019
  Multiple errors in logs. It looks like calls were during 1:45 AM to 6:00 AM. During the period 1:45 AM to 6:00 AM, all messages are failing due to Clarity Refresh.
- November 27, 2019
  Server reboot schedule, Control Tower team was not notified of schedule leading to unexpected downtime.
- July 24, 2020
  Increase in FHIR (Fast Healthcare Interoperability Resources) API (application programming interface) for real-time observation calls leading to timeouts of model predictions.
- February 15, 2021
  Generic FHIR API error call: “HTTP error code: 500.”
- April 8, 2021
  Model errors after Epic upgrade.
- July 30, 2021
  Troponin issues fixed causing covariate drift in model scores.
- September 15, 2021
  Production system competed for resources requiring scale back of Control Tower scores updates. Errors created and schedule has now been updated for processing.
- January 5, 2022
  Nightly Clarity Database delay causing morning score errors.
- May 16, 2022
  IBM queue server certificate update causing server errors.
- November 7, 2022
  Data mart for diagnoses codes update causing pipeline to break down.
- November 8, 2022
  Issues with Clarity Database slowing down Control Tower queues.
- March 21, 2023
  Control Tower FHIR API for real-time unit changes failing for a single request, causing payload slowdown.
- April 5, 2023
  JSON structure changing causing model error (unintended repo change).
- May 1, 2023
  An unplanned issue impacting Enterprise API Services, who manages real-time data feeds, resulting in internal server error.

### Monitoring Protocol

**Figure 7:**
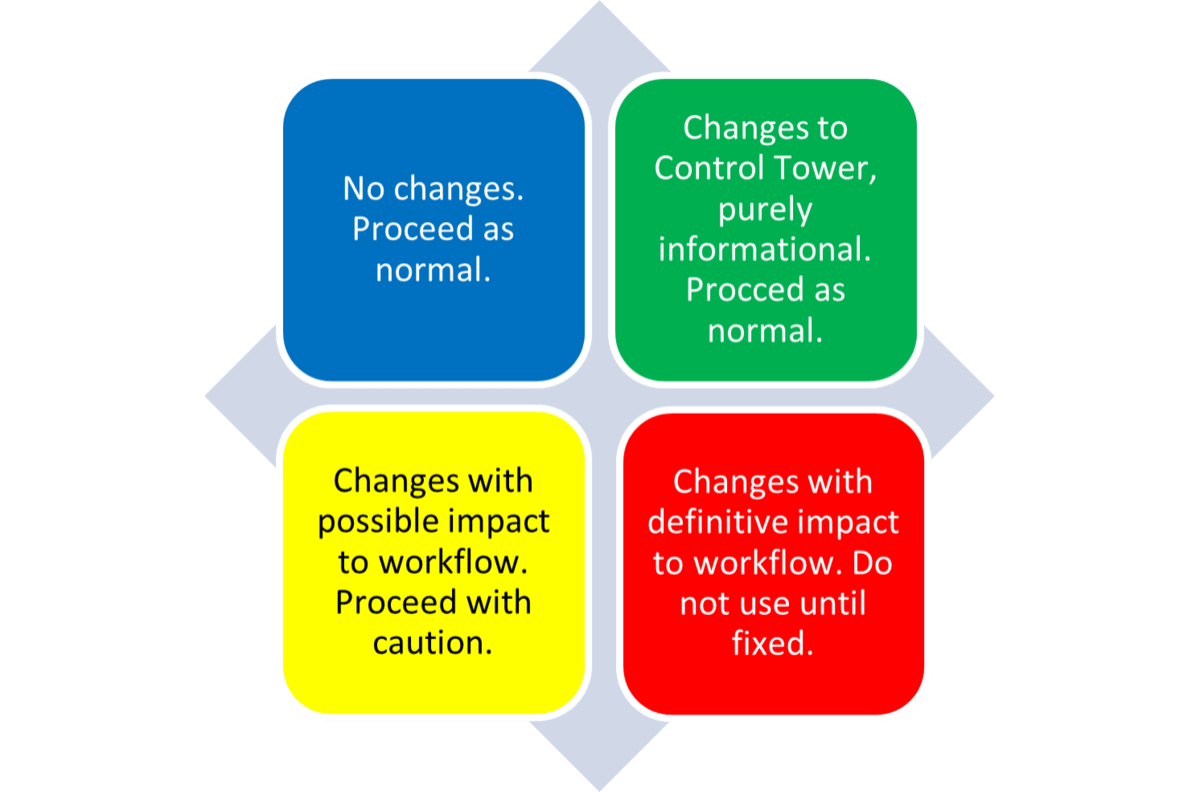
Communication triage protocol for Control Tower. The protocol’s stages are color-coded to signify different statuses and recommendations: blue for normal operation, green for minor layout changes, yellow for potential performance issues, and red for significant errors.

Actions prompted by model monitoring feedback were synthesized into a protocol to communicate model failures and downtimes with clinical staff. The Control Tower protocol used a triage system, consisting of 4 stages in which each stage prompts a message to the clinical team, as outlined in Figure 7. The first stage (blue) was reserved for when everything was operating as expected. Stage 2 (green) indicated a minor change in the layout of the tool, such as a user interface change. These first 2 stages delivered informational prompts to the user, notifying them of the tool’s status and requiring no action from the user. The third stage (yellow) indicated possible performance degradation, such as when patient scores from the model were not up-to-date, that is, a day old. The day-old scores can still provide evidence for action, but the clinical team may need to be cautious, as updated scores can change the patient’s risk. As such, users were notified of these issues and asked to use the tool at their discretion. The last stage (red) indicated a significant error within the tool, such as a covariate completely missing from the model’s input. This stage would notify staff not to use the tool until a fix was implemented.

## Design Considerations

### Overview

From our experience of developing and implementing the Control Tower monitoring platform, we have derived a series of broader considerations necessary for model monitoring to serve as generalized guidelines for future implementations. Central to these guidelines arose themes of feasibility, design, implementation, and policy. While existing frameworks have proven successful in managing long-term IT infrastructure projects in health care, ML models are experimental and inherently open systems, entailing costly development and maintenance. As such, they demand additional considerations due to their reliance not solely on technical data but also on statistical and clinical assessments to identify errors. Consequently, there is no unequivocal, predetermined signal that can be provided to an IT group lacking clinical or data science expertise to detect these errors. Identifying them often necessitates the accumulation of statistical data over time. While readily available and accessible data may be used to identify some errors, others may require weeks or months of new data collection to draw inferences. Furthermore, in traditional software operations, refinements can be implemented more quickly. Responding to user feedback can often be met with bug fixes or minor feature requests. However, implementing refinements to an ML model often requires a longer development cycle, as many changes will require a complete retraining of the model or the acquisition of novel data. Such complications in model maintenance underscore the need for input from multiple teams, alongside established structures and policies, to ensure effective orchestration of model maintenance.

### Feasibility: Are the Resources to Facilitate Productive Monitoring Available?

Successful real-world implementation of models must consider the present and *future* bandwidth limitations of a team. In an ideal scenario, a team would be provided ongoing dedicated time or personnel to ensure the upkeep of deployed models. However, this is not always feasible, and the responsibility of maintenance competes with a team’s constant stream of new projects and tasks. As such, it is important to first determine how long-term monitoring of a model (or eventually, *models*) will be integrated into the team’s workflow. For example, who will check in on the model and with what frequency? If and when the model requires retraining, who will perform the retraining, and how will they be guaranteed the flexibility to shift from their current projects to accomplish this?

In addition to personnel, computational resources must also be considered for long-term monitoring. Regarding storage requirements, the amount of data produced from monitoring depends on a variety of factors, including the model’s feature set, the frequency of calls made to the model, and the number of feature and performance metrics that will be tracked. For example, a model that delivers constant, real-time feedback in a critical care setting may, in turn, require constant monitoring to ensure performance does not suddenly degrade, resulting in performance metrics and feature distribution logs needing to be generated continuously.

When assessing the feasibility of long-term monitoring, an attractive option to consider is automated monitoring: developing models that detect whether a significant change has occurred in a deployed model’s predictions or its incoming data. While our team chose a “hands-on” approach, others have found success in implementing an additional model for performance monitoring to notify the team of data shifts and, in some cases, automatically retrain the original model [16]. Ultimately, the decision to use an automated monitoring model comes down to the type of model deployed, the bandwidth of the team, and the reliability of the data to support automatically retraining the deployed model. In any case, even an automated monitoring model will still require some human monitoring as well. Our team is currently working on an automated monitoring system for this purpose.

### Design: Deciding What and How to Monitor?

#### Overview

The design of an investigative platform to facilitate model monitoring may range from dynamic and interactive interfaces to static reports, the choice of which is dependent on feasibility factors and nuances of the particular scenario, but design should ultimately enable rapid and comprehensive assessment. Furthermore, there are standard functionalities each platform should feature to appropriately assess long-term model performance.

Deployed models encounter performance declines through distributional and relational shifts in the underlying data [17]. These shifts are the crux of why postdeployment monitoring is necessary, and no model is immune to them, regardless of how well it performed in its testing environment [18]. This impediment has received a wealth of attention from the ML community and has been synthesized into 3 types of distinct shifts.

#### Prior Probability Shift

*The distribution of the target variable Y changes between the training data and incoming data, but not P (X|Y)* [19]. This can occur when the prevalence of a disease changes over time in the target population; however, the underlying factors that cause the disease remain constant, for example, a spike in influenza rates during the influenza season. Prior probability shift is assessed by monitoring the distribution of the target variable over time, measuring for any sudden or gradual changes.

#### Covariate Shift

*Distributions of input data diverge between training data and new incoming data* [4]. Such shifts may occur in the clinical setting, for example, when diagnostic screenings are updated. This procedural change may decrease the specific laboratory values, which are heavily relied upon by the model. Conversely, diagnostic variables that were initially infrequent may become more prevalent over time. This can result in situations where the model, which had limited instances of these variables during training, struggles to fully capture, and therefore use, their predictive signals.

#### Concept Drift

The relationship between the incoming input data and target variable changes over time, drifting from the original relationship captured in the training data [20]. The COVID-19 pandemic provided a real-world example of concept drift, as hospital census models were affected by admissions that drastically moved toward higher-risk patients due to increases in complications from the COVID-19 disease and decreases in hospital use among people with a milder spectrum of illness [21].

#### Usability

A successful UI will take into consideration the professional backgrounds of those using the platform. However, when the responsibilities of monitoring are handed to a different group, the new group’s level of familiarity with the model should guide the design. For example, guidelines for what is acceptable variance should be established and implemented. One method for accomplishing this may be through using control charts, allowing the modeling team to prespecify a simple and visual approximation of how much drift is tolerable before action must be taken [22].

### Implementation: How Will the Platform Be Built and Sustained?

When implementing a monitoring platform, it is necessary to consider how the back end of the platform will process and store the necessary data elements. The efficiency of this task is critical and must accommodate the model’s scale and responsiveness. Data can amass quickly as large feature sets are monitored, and the model may be called frequently to predict on many patients throughout the day. Furthermore, the back end must be capable of efficiently parsing, formatting, and, if necessary, compressing the data into clean data sets for the platform to analyze and visualize. For Control Tower, many of these data storage requirements were already in place for capturing and storing the necessary patient elements. This will likely be the case for many clinical scenarios, as patient data must be securely and efficiently housed. Instead, implementation efforts are more apt to center around ensuring these data elements are efficiently piped to the monitoring platform.

Using a web application for model monitoring provides a dynamic interface, allowing any user with log-in permissions to view the real-time status of the model and the surrounding data. This investigation mechanism can eliminate potential confusion, which may arise from a routine generation and sharing of static technical reports, such as accidentally referencing outdated documents. When selecting a programming language to build the app, preference should be given to those languages that facilitate efficient app development. For Control Tower, R Shiny was used given the team’s previous experience with the package and strong background in R. The R package provides a user-friendly environment for quickly creating, testing, and publishing web applications. Similar web application tools exist across multiple programming languages including Python and Java, and as such, teams are likely to find a web application package in a language they are familiar with.

When coding the app, modular coding practices should be adopted to ensure flexibility and scalability. Such adoption promotes versatility of the app to incorporate additional statistical measures or visualizations and allows the app to be easily translated for other monitoring use cases. Leveraging modular coding practices at the onset of app development allows for future additions, revisions, and ports to be made with minimal effort. For Control Tower, modular coding practices were primarily used to better facilitate development across multiple team members. This practice allowed for functions to be easily repurposed by other team members to avoid duplication of work and to allow the app to be easily extended to other ML models within Mayo Clinic.

The number of programming languages used in the data pipeline plays a significant role in shaping the development process and the overall efficiency of the monitoring. To facilitate this, minimizing the number of programming languages used across the various tasks can streamline development and maintenance through ease of interpretability and integration. This can reduce maintenance costs and overhead by reducing interoperability concerns and decreasing the learning curve for new team members. Minimizing these ongoing costs is a necessity when considering the model will ideally be in production long term. However, if the development team is proficient in multiple languages, leveraging the strengths of each may have its advantages, such as reducing bottlenecks in development or data transfer, while increasing the flexibility of a system. In the case of Control Tower, R (R Foundation for Statistical Computing), Python (Python Software Foundation), and shell scripts were used, favoring R for app development, Python for data processing, and shell scripts for scheduling various model and platform tasks.

In addition, upstream problems will inevitably manifest; therefore, implementing a notification system for these errors can proactively address disruptions, minimizing the downtime of the pipeline. One method for accomplishing this is to incorporate error logging and alerts into cron jobs, which can immediately notify the team of any failures. Such notifications are critical for model monitoring, as some errors may be undetectable to the end user, resulting in the continued use of inaccurate information. As such, it is vital for monitoring teams to identify, communicate, and resolve errors as soon as possible.

Finally, integrating regular checking of the platform into the team workflow allowed the team to not only stay abreast of model performance but also maintain an intuitive sense of potential broader complications surrounding the model. For example, monitoring the probability distributions of the model ultimately provided the team with a sense of whether further investigations into the model would be necessary. However, investigations into the distributions of the individual features allowed for potential diagnoses as to why the model may begin to degrade in performance, as well as alluded to data pipeline errors that may be present. By maintaining a sense of these wider issues, shifts in the outcome could be more easily prevented and diagnosed

### Policy: What Is the Response to Platform Feedback?

#### Overview

Once the monitoring platform is deployed and available, the next stage of considerations surrounds how knowledge provided by the platform will be used. A set of policies must be developed to determine which actions will be taken based on monitoring feedback, addressing such questions as “At what point is a data shift significant enough to prompt retraining?” and “How will errors be communicated with technical and clinical staff?” Generally, such a policy should cover error designation and response, when to retrain, and how to communicate failures. In addition, a well-defined policy allows for the task of monitoring to more easily be extended across various teams and roles.

#### Error Designation and Response

It is essential to establish and define a process that determines when a specific degree of shift or drift in the model qualifies as an error warranting a response. The question *“How much drift is necessary to take action?”* represents one of the more subjective aspects of model monitoring. In scenarios where multiple team members are tasked with overseeing model performance or possess limited familiarity with the model, substantial interrater variability becomes a concern. For example, one team member might observe a 5% shift in the distribution of a feature and consider it inconsequential, while another member might view it as a reason for immediate action. To address this variability, the Control Tower team would send email updates to other team members detailing any shifts that were noticed; this would allow for a collective discussion on whether to take action as well as allow for a convenient forum to keep all team members updated on the model’s status. Regardless of the criteria used to identify shifted covariates or outcomes, team members must communicate and establish agreement on the minimal drift threshold requiring action, while ensuring that utmost priority is placed on maintaining optimal model accuracy.

Even with consensus on the magnitude of a shift, several contextual factors can influence the team’s risk tolerance toward these shifts. Significant changes may occur without sustained trends, indicating a regression to the mean. Alternatively, a dramatic shift might happen for a variable with minimal contribution to the risk score. While predefined cutoff points could be considered to standardize investigations, these benchmarks may still necessitate ongoing human review and could vary for each feature, making it impractical to define for every feature in large feature sets.

Even if an error is defined with a certain level of risk in mind, there are considerations in the response to the error and the amount of time one needs to allocate for remediation. A deployed model is prone to errors from a variety of sources, ranging from data shifts to IT scheme modifications. Given the diversity of potential errors, an effective policy will include guidelines for the categorization of errors along with the appropriate responses to each. The errors encountered with Control Tower fell broadly into 4 categories.

1. Technical infrastructure: database issues, expiration of certificates, and password updates often causing the pipeline to fail
2. Explained shift: a significant data shift with an identified root cause
3. Unexplained shift: a significant data shift with an unidentified root cause
4. Performance loss: a decrease in the model’s performance metrics, which may manifest with or without data shift

Categorizing errors for appropriate response is crucial, as it establishes a standardized knowledge base for reporting, ultimately enhancing the efficiency of troubleshooting. Categorization often leads to the discovery of similar strategies for mitigating similar error types. For instance, errors related to database refreshing or password expiration typically do not require immediate intervention, while performance losses in accuracy or calibration often necessitate retraining of the model. Appropriate categorization also offers the advantage of reducing risk tolerance while enhancing response efficiency. Having encountered an error previously increases the likelihood of streamlining investigations, enabling the examination of lower-risk shifts or drifts.

When ongoing outcomes data are available, performance loss can be detected by looking for significant shifts across a variety of classification performance metrics including area under the receiver operating characteristic, area under the precision-recall curve, calibration, subgroup differences, and so on. When such data are absent, as in the case of Control Tower, performance loss can only be inferred by looking for significant shifts in the distribution of predicted probabilities of the model. To supplement assessing predicted probabilities, potential performance loss may also be identified by looking for significant shifts in the features of the model. While significant shifts may occur in these features without significant shifts in the model’s output, drifts in feature distributions can signal other potential problems necessary to address. While performance loss may be resolved or mitigated through upstream pipeline errors, some instances may require the model to be retrained.

#### Model Retraining

The circumstances for when to retrain a model will vary across teams and platforms, often dependent on the cost of retraining. As such, it is necessary for a platform policy to clearly state when, and when not, to retrain. For example, many errors will not require model retraining such as simple pipeline errors or data shifts due to changes in medical coding, requiring only a small update to the pipeline. Therefore, it is important to first identify and fix any upstream errors before considering retraining. There are even instances of significant shifts that do not warrant retraining. For example, one could have several shifted covariates in the model with trivial importance scores, effectively having no impact on predictive performance. From the perspective of model importance, one may bin covariates that have little impact and essentially treat them as nuisance variables.

Assuming no upstream errors are present, a model should always be retrained when significant and sustained performance loss is encountered. Defining *significant* and *sustained* will be specific to each scenario, depending on the algorithm and health care delivery model. However, it is incumbent upon the team to define an appropriate window for performance to vary, with a lower limit triggering retraining.

It is important to note that retraining does not have to be used sparingly, assuming the bandwidth is available. When feasible, it may be good practice to routinely retrain the model with the expectation that updated data are more current with clinical practice. Such versioning of the model would allow for new features, incremental improvements, and technical debt management. For Control Tower, versioning allowed us to spot potential bugs or fixes and investigate new features.

#### Communicating With Clinical Staff

The clinical team using the model’s outputs must be consistently informed about the model’s status due to its significance to their workflow and overall trust in the model. The model’s standard operating procedure outlines how the clinical team should use the model and details communication protocols between IT, data science, and the clinical users. Protocols should consist of dedicated contacts for various issues and plans for how to operate during model performance shifts and downtime.

## Discussion

### Principal Findings

As ML models require consistent monitoring to ensure sustained accuracy, a series of decisions must be made for how best to integrate model monitoring into a team’s workflow. Problems, considerations, and solutions that arise from this process can vary greatly depending on the setting, nature of the model, and available bandwidth, both from the technical team and their computational resources. While prior work has established the importance of monitoring and corresponding statistical solutions, this paper provides specific considerations and solutions derived from the real-world implementation and day-to-day use [23,24]. Throughout the integration of Control Tower, our team found that these considerations centered around 4 phases that serve as a road map when planning a long-term modeling strategy: feasibility, design, implementation, and policy.

### Experiences

Development and implementation of the platform faced several obstacles, which we attribute to the inherent realities of integrating real-world applications. First, the team was unable to complete the platform by the time the associated clinical trial for the Control Tower model began recruitment. This required the team to omit crucial features from the platform, such as monitoring for concept drift. Monitoring concept drift required collecting ground truth outcomes, that is, whether patients actually received palliative care. Collecting these patient outcomes required building a separate data pipeline, which was the team’s original intent, but as the team took on additional tasks, the pipeline was passed over in favor of monitoring predicted probabilities. While omitted from Control Tower in scenarios where outcomes data are tracked, we direct the readers to literature providing a more comprehensive understanding of concept drift [25-27].

The original intention for Control Tower was to have the model run any time a patient’s laboratory values were updated, ensuring that the patient’s risk scores were always reflective of current data. While the model was originally deployed using this dynamic system, it, unfortunately, proved too taxing for the IT infrastructure in which it was hosted. To alleviate this problem, the model and platform were switched to running on a batch schedule, updating patient risk scores and the monitoring platform every 4 hours. While this delivery schedule proved more manageable, model calls made between these 4-hour updates ran the risk of using outdated patient data, potentially impacting performance. Given that the workload imposed by the original schedule was infeasible, this was considered a fair compromise. Finally, the implementation of the platform occurred during the COVID-19 pandemic, which affected staffing and resulted in IT furloughs. Unfortunately, this meant that technical infrastructure problems, which could typically be fixed by IT on the same day, instead, took up to 1 week to fix, resulting in prolonged downtime for the model.

Despite these challenges, there were several positive experiences to highlight. First, a significant amount of collaboration occurred within the data science team in order to have the monitoring platform in a usable state by the time of the model’s clinical trial. This required analysts to tend to a variety of tasks, often on a moment’s notice. Following deployment, there was also sufficient bandwidth from the team members to continue monitoring the platform as they took on additional projects. Second, IT furloughs as a result of the pandemic were resolved within 6 months, allowing routine technical infrastructure issues to once again be resolved on the day of occurrence, resulting in less model downtime. Finally, the model’s predicted probabilities remained, for the most part, consistent, making for a stable tool throughout the documented 2 years of use. Using a simple linear regression model, we examined the relationship between daily predicted probabilities (dependent variable) and time since deployment (independent variable), observing a slope of 0.005 at a *P* value of <.001, suggesting a statistically significant, but functionally small trend, with the mean probability increasing .005% each day.

### Limitations

Despite a thorough detailing of our experiences, it is important to note that this paper covers only a single implementation. While we have recounted the challenges and considerations necessary for Control Tower, this is not an exhaustive list, and other teams and platforms may encounter challenges foreign to ours.

### Future Considerations

The Control Tower platform allowed our team to successfully monitor performance and maintain our deployed model for 2 years. Moving forward, our team is planning to automate parts of this task, for example, by implementing an automated email notification system, notifying the team when the number of model calls, predicted probabilities, and incoming data streams shift beyond their respective significance thresholds. While this modification is not intended to outright replace the manual checks of the platform, it will allow the team to check the platform at a lesser frequency. This system will serve as a placeholder while the team develops a new model to monitoring the performance of Control Tower, leveraging supervised learning to detect shifts in the probability and multivariate covariate distributions [28,29].

The team also considered an online or continuous learning model to automatically address data drift. In continuous learning, the algorithm would update its predictions as new data come in and alleviates the need to manually retrain the data [30]. Although appealing, an automated system, in this sense, would require more policy changes and would bring with it a number of issues. First, there are several cost and computing issues that could make an implementation difficult, as entire systems would need to know when to train and to do it without interrupting the current pipeline, as well as a validation step to ensure sustained, if not improved, accuracy. Second, the algorithm must remain trustworthy for clinicians. Did the algorithm *unlearn* anything important? Did it learn anything irrelevant or incorrect? As an example, if a covariate shift occurred due to a missing laboratory code, resulting in increasingly missing values of that laboratory, we would not want the model to learn a new relationship with the missingness; instead, we would make an update to the data pipeline to resolve the missingness. Finally, all continuous learning models require ready access to the gold-standard outcome, which might not be feasible in all cases.

### Conclusions

Once an ML model has been successfully developed and deployed, it must be continuously monitored to ensure its efficacy amidst an ever-evolving practice and stream of patients. While a variety of methods have been proposed to statistically monitor the performance of models, this is only one factor to consider when implementing a long-term modeling strategy. By disseminating the broader experiences of integrating ML monitoring platforms into clinical practice, readers will be better equipped for the considerations and challenges encountered during their own implementations.

## Appendix

Multimedia Appendix 1 Source code for a demo of the Control Tower Model Monitoring R Shiny application.

## Abbreviations

GUI: graphical user interface
ML: machine learning

## Data Availability

The data sets generated during and analyzed during this study are not publicly available due to concerns of patient identification but are available from the corresponding author on reasonable request. Such requests may require separate approval by the Mayo Clinic Institutional Review Board.
